# Initiation and stability of self‐harm in adolescence and early adulthood: investigating social and aetiological factors in twins

**DOI:** 10.1111/jcpp.14096

**Published:** 2024-12-13

**Authors:** Filip Marzecki, Yasmin I. Ahmadzadeh, Olakunle A. Oginni, Jean‐Baptiste Pingault, Thomas A. McAdams, Helena M. S. Zavos

**Affiliations:** ^1^ Institute of Psychiatry, Psychology & Neuroscience King's College London London UK; ^2^ The Wolfson Centre for Young People's Mental Health Cardiff University Cardiff UK; ^3^ Division of Psychology and Language Sciences UCL London UK; ^4^ PROMENTA Research Centre University of Oslo Oslo Norway

**Keywords:** Self‐harm, twins, aetiology, age of initiation, bullying, socio‐demographic factors

## Abstract

**Background:**

Almost one in five (18.8%) UK adolescents are estimated to self‐harm and many young people initiate self‐harm early (average age 13 years). Prevention of self‐harm should be informed by knowledge about risk factors (e.g. socio‐demographic indices), characteristics (i.e. motivation for self‐harm and help‐seeking behaviours), as well as relative aetiological genetic and environmental processes. Previous twin studies evidence both genetic and environmental influences on self‐harm. However, to date, there has been no genetically informed research on self‐harm aetiology across development, nor studies identifying risk factors for initiating self‐harm at a younger age.

**Methods:**

We examined self‐harm in the Twins Early Development Study, a birth cohort twin study. Using clustered regression models, we tested associations of socio‐demographic factors and victimisation with lifetime self‐harm and age of self‐harm initiation, both reported at 21. To investigate stability and/or change in genetic and environmental influences on self‐harm we interpreted a multivariate Cholesky decomposition across ages ≤16, 21, and 26.

**Results:**

Self‐harm was more common in adolescence than early adulthood, and the incidence of self‐harm in early adulthood was low (1.4%). The most common motivation for self‐harm was ‘to get relief from a terrible state of mind’ (83.4%). Independent predictors of self‐harm and earlier initiation of self‐harm were being female, belonging to a gender and/or sexual minority group, and experience of bullying victimisation. Sexual minority status was still significantly associated with self‐harm after controlling for familial factors in co‐twin control analyses. The Cholesky decomposition showed stability in genetic influences and innovation in non‐shared environmental influences on self‐harm.

**Conclusions:**

Adolescence should be a key period for self‐harm interventions. Women, sexual, and gender minorities, and those experiencing victimisation may need targeted support early in adolescence. Furthermore, it should be acknowledged that different individuals can be at risk at different stages as environmental factors influencing self‐harm change across time.

## Introduction

Self‐harm is an act of injuring or poisoning oneself ‘irrespective of the apparent purpose’ (National Institute for Clinical Evidence, 2022). Recent evidence suggests that suicidal and non‐suicidal self‐harm share similar aetiologies as well as strong associations with depressive symptoms and might be part of a spectrum rather than two discrete entities (Lim et al., 2020). Self‐harm is particularly prevalent during adolescence with almost one in five UK adolescents estimated to self‐harm (Kidger, Heron, Lewis, Evans, & Gunnell, 2012) and suicide is amongst the most common causes of young people's deaths worldwide (WHO, 2021). Prevention of self‐harm should be informed by knowledge about characteristics (i.e. motivation for self‐harm and help‐seeking behaviours), risk factors (e.g. socio‐demographic), and aetiological genetic and environmental processes so that interventions can target these mechanisms. Nonetheless, there is less research on risk factors and characteristics specifically associated with the initiation of self‐harm at a younger age, as well as a lack of genetically informed research relating the age of initiation to the aetiology of self‐harm.

In the first birth cohort study of adolescent self‐harm in the UK, lifetime prevalence was 18.8% amongst 16–17‐year‐olds, and 5.7% of participants self‐harmed with suicidal intentions (Kidger et al., 2012). Research indicates that self‐harm tends to be initiated in early adolescence. For example, in a UK community‐sample of nearly 4,000 adolescents, 13% of 12‐ to 14‐year‐olds reported concurrently self‐harming (Stallard, Spears, Montgomery, Phillips, & Sayal, 2013). A meta‐analysis of studies conducted on adolescents with a mean age of 15.4 in 41 countries found the average age of self‐harm initiation to be 13 years (Gillies et al., 2018). Studying the factors associated with early initiation matters because early initiation is associated with greater severity of later self‐harm (Ammerman, Jacobucci, Kleiman, Uyeji, & McCloskey, 2018) as well as with later suicidal ideation and suicide (Somer et al., 2015). Understanding more about factors associated with initiation will also help identify individuals who should be targeted by early interventions.

There is variability in motivations for self‐harm. A recent meta‐analysis of studies on adolescents in 41 countries found that the most commonly endorsed motivation for engaging in self‐harm was ‘to obtain relief from feelings or thoughts’, and ‘to punish oneself’ (Gillies et al., 2018). Young people also vary in seeking support, with previous research suggesting that most do not seek help for their self‐harming (e.g. Rowe et al., 2014; Stallard et al., 2013). Those that seek support, most commonly do so from friends, then family (Gillies et al., 2018). Only 8% of adolescents who self‐harm sought medical help, as found in the meta‐analysis (Gillies et al., 2018).

In addition to the characteristics of self‐harm, socio‐demographic and environmental risk factors should be considered when working to prevent self‐harm. These factors include parental socioeconomic status (e.g. Engström, Diderichsen, & Laflamme, 2004; Page et al., 2014), female gender (Gillies et al., 2018; Plener, Schumacher, Munz, & Groschwitz, 2015), being a sexual minority (i.e. not heterosexual, e.g. Oginni, Robinson, Jones, Rahman, & Rimes, 2019), and a gender minority (i.e. non‐binary or transgender, e.g. Rogers & Taliaferro, 2020), as well as experiencing bullying victimisation (Fisher et al., 2012; Islam, Khanam, & Kabir, 2020). In previous genetically informed researched, and more specifically in co‐twin control studies, some of these factors, namely, sexual minority status and bullying, have been associated with self‐harm suggesting likely causal relationships (Baldwin et al., 2019; O'Reilly, Pettersson, Quinn, et al., 2021; O'Reilly, Pettersson, Donahue, et al., 2021). The nature and the rates of self‐harm also differ by ethnicity in the UK, with rates being lowest amongst Asian men and highest amongst Black women as reported in systematic reviews on a mixture of adolescent and adult samples (Al‐Sharifi, Krynicki, & Upthegrove, 2015; Bhui, McKenzie, & Rasul, 2007).

Genetic influences have been implicated in self‐harm aetiology (e.g. Voracek & Loibl, 2007; Lim, Krebs, Rimfeld, Pingault, & Rijsdijk, 2022). Research amongst young adults found that genetic variance explained respectively 50% and 55% of the variance in both suicidal and non‐suicidal self‐harm, evidencing moderate genetic influences. Genetically informed research on self‐harm can help to highlight socio‐demographic and environmental risk factors, because it allows us to separate genetic influences from environmental influences, in turn highlighting the relative role played by the latter. To date, there have been no studies investigating the aetiology of self‐harm over development.

Using data from a longitudinal population‐based sample of twins – the Twins Early Development study (TEDS), we aimed to (1) estimate the prevalence of self‐harm at three time points (≤16, 21, and 26 years of age); (2) explore the socio‐demographic and environmental factors associated with self‐harm and age of initiation of self‐harm; (3) investigate the relative genetic and environmental influences on self‐harm across adolescence and young adulthood.

## Methods

### Participants

Data from Twins Early Development Study (TEDS) were used – a cohort study of twins born in England and Wales between 1994 and 1996 (Lockhart et al., 2023). We analysed data reported by parents at first contact, as well as data reported by twins around ages 12 (*M* = 11.28; *N* = 11,728), 14 (*M* = 14.07; *N* = 6,635), 16 (*M* = 16.48; *N* = 5,237), 21 wave 1 (*M* = 22.27; *N* = 10,301), 21 wave 2 (*M* = 22.85; *N* = 8,860) and 26 (*M* = 26.38; *N* = 7,827). The two phases of data collection at age 21 included different measures and took place roughly 8 months apart. The study received full ethical approval from the Health Faculties Research Ethics Subcommittee at King's College London (05/Q0706/228) and the TEDS team obtained informed consent when twins were contacted for each wave of data collection. Participants were excluded from analyses as per standard medical exclusion criteria for the cohort: having a severe medical condition, having experienced severe perinatal complications, or having unknown zygosity. Zygosity was assessed using a parent‐reported measure of physical similarity during childhood which, compared with DNA testing, had 95% accuracy (Price et al., 2000).

TEDS is relatively representative of the UK socioeconomic spectrum (Rimfeld et al., 2019). Relative to UK general population estimates, the latest data collection wave of TEDS (population born 1994–1996) had a larger proportion of women (64.5% vs. 51.0%), a similar proportion of transgender people (0.6% vs. 0.5%), a larger proportion identifying as a sexual minority (14.8% vs. 3.2%), and a smaller number of racialised minority people (7.3% vs. 18.3%). See Table S1 for detailed descriptive statistics of the sample.

Participant attrition is a concern in longitudinal studies, for example, those who self‐harm and have poorer mental health may be less likely to respond to data collection requests at later points. We found that self‐harming at ages ≤16 and ≤21 was positively associated with responding to the subsequent data collections, but only weakly (see Table S2).

### Measures

#### Self‐harm

Self‐harm was self‐reported at ages 21 (phases 1 and 2) and 26. The questions were adapted from the Child & Adolescent Self‐Harm in Europe (CASE) study (Madge et al., 2008). Self‐harm was treated as a dichotomous variable for the descriptive statistics and twin models, but both as a dichotomous and a continuous variable for regression models.

##### Lifetime self‐harm (age 21)

The twins were asked about self‐harm across their lives at 21 (phase 1): ‘In your lifetime, have you ever hurt yourself on purpose?’. Five options were listed for them to respond, which were ‘No, never in my lifetime’, ‘Yes, once or twice’, ‘Yes, 3–5 times’, ‘Yes, 6–10 times’ and ‘Yes, more than 10 times’ coded as ‘0’ to ‘4’ respectively. For dichotomous analyses, those responding ‘No, never in my life’ were coded as ‘0’ and the rest were coded as ‘1’.

##### Self‐harm (ages 21 and 26)

The twins were also asked about self‐harm in the preceding 12 months at ages 21 (phase 2) and 26: ‘In the past 12 months, have you ever hurt yourself on purpose?’ with the same response options and continuous/dichotomous coding as for the lifetime self‐harm question.

##### Motivations for self‐harm and help‐seeking

At age 21 (phase 1), as part of the lifetime self‐harm questionnaire, the twins rated different motivations for self‐harm on a 5‐point Likert scale from *not at all* to *very much*. These were: ‘to show desperation’, ‘to die’, ‘to punish oneself’, ‘to frighten someone’, ‘get relief from a terrible state of mind’. For the descriptive statistics, responses to the motivation for self‐harm questions were dichotomised with ‘not at all’ and ‘not really’ coded as ‘0’ and the remaining answers coded as ‘1’. They were also asked if they ever sought medical help from GP, A&E, or a different healthcare professional after hurting themselves. These responses were recoded into help‐seeking from any healthcare professional – those who reported seeking help from at least one source were coded as ‘1’ and those who did not seek help from any source, were coded as ‘0’.

##### Age of initiation of self‐harm

At age 21, twins reported retrospectively the age at which they first initiated self‐harm, with options being: 11 or younger, 12, 13, 14, 15, 16, 17, 18 or older.

##### Self‐harm ≤16

The TEDS data collection waves in adolescence did not ask about self‐harm, hence a retrospectively reported variable was computed, ‘Self‐Harm at ≤16’, from the lifetime self‐harm question at 21 and the age of initiation question. Those who responded ‘Yes’ (any frequency) to the lifetime self‐harm question as well as reported initiation at age 16 or younger were coded as 1, and the rest were coded as 0, except for missing data. We selected 16 and younger, as this reflects the period of compulsory secondary education in England and Wales for participants in this sample.

#### Socio‐demographic factors

##### Socioeconomic status (SES)

At first contact, maternal and paternal employment status and educational levels were reported, and a composite score was obtained (a sum of standardised responses to each of these questions), which was used as an index of socioeconomic status (SES). The composite score was standardised again after the summing up (Hanscombe et al., 2012).

##### Ethnic background and racialised minority status

Racialisation emphasises the socially constructed nature of race (Hochman, 2019) – as such, we refer to ‘racialised’ minorities rather than ‘ethnic’ or ‘racial’ minorities. Ethnic identity was reported at first contact by twins' parents. The categories available to respondents were: Asian, Black, White, Mixed Race, Other, with a text box if ‘Mixed’ or ‘Other’ were selected. Ethnic identity at first contact was dichotomised into white and non‐white, with the latter extrapolated to represent racialised minority status.

##### Sexual orientation and sexual minority status

At age 26, twins self‐reported their sexual orientation, with the following options: heterosexual, homosexual, bisexual, pansexual, asexual, fluid, prefer to self‐define, unsure/I don't know, and prefer not to answer. These were used for the descriptive statistics, but for the inferential analyses, the scores were dichotomised into ‘heterosexual’ and ‘sexual minority’. All participants responding ‘heterosexual’ were coded as 0, and all the remaining participants were coded as ‘1’, except for those responding ‘unsure/I don't know’ and ‘prefer not to answer’ who were removed from analyses. Participants who responded ‘prefer to self‐define’ to the sexual orientation question were coded as missing, because there was no option to write in self‐identification in the questionnaire, leaving insufficient information to conclude which of these participants belonged to a sexual minority. For example, self‐identification of gender and sexuality can lead to understanding errors, such as a respondent identifying as heterosexual choosing to write a self‐identification responses and writing ‘straight’ instead of selecting ‘heterosexual’.

##### Gender identity, trans identity, and gender minority status

At age 26, participants reported their gender identity (male, female, non‐binary/gender‐queer, prefer to self‐define, don't know, prefer not to answer) and whether they identified as transgender (yes, no, prefer not to answer). Out of the responses to the gender and transgender identity questions, a gender minority status variable was computed, whereby those responding ‘male’ or ‘female’ to the gender question and ‘no’ to the transgender question were coded as 0. Those responding ‘non‐binary/gender‐queer’ to the gender identity question and/or ‘yes’ to the transgender question were coded as 1. For 21 participants who responded ‘prefer to self‐define’ to the gender identity question were coded as missing, because there was no option to write in self‐identification in the questionnaire, and self‐identification of gender and sexuality can lead to understanding errors, for example, a respondent identifying as a woman choosing to write a self‐identification response and writing ‘woman’ instead of selecting ‘female’. A participant who does that would not belong to a gender identity, despite being coded as ‘prefer to self‐define’.

##### Lifetime victimisation

Bullying victimisation was self‐reported by twins at ages 12, 14, 16, and 21. At ages 12 and 14, twins completed the Multidimensional Peer Victimisation Scale (MPVS; Mynard & Joseph, 2000). At age 16, they completed a shortened version of the MPVS, with six items only. At age 21, twins responded to an adapted version of the Multidimensional Peer Victimisation Scale‐Revised (MPVS‐R; Betts, Houston, & Steer, 2015). The exact items on each peer victimisation scale used can be found in Appendix S1. A lifetime victimisation score was obtained by standardising victimisation scores at ages 12, 14, 16, and 21 and creating a sum of these, with higher scores on this measure indicating greater severity as well as the chronicity of victimisation. Missingness was dealt with by including participants who completed victimisation scales on least three out of four ages considered.

### Analyses

#### Phenotypic analyses

To estimate the prevalence of self‐harm across time, percentage frequencies were obtained for dichotomous self‐harm reported at ages ≤16, 21, and 26. To test group differences, Chi‐square tests were conducted to examine the differences in self‐harm and age of initiation between racialised groups, gender identities, and sexual identities.

To explore the socio‐demographic and environmental factors associated with self‐harm and age of initiation of self‐harm, logistic (binary outcomes) and linear (continuous outcomes) regression models were specified with SES, female gender, gender minority status, sexual minority status, and lifetime victimisation as independent (or predictor) variables and self‐harm (lifetime, reported at age 21) as the dependent variable. For logistic regressions with a binary self‐harm outcome, where the predictor variable was continuous, results are reported in terms of odd ratios, which compare individuals who differ by one unit of a continuous predictor. When predictors are categorical, odds ratios compare individuals at a particular level of the predictor to a reference level. Unadjusted models are given the main interpretative weight to avoid the ‘table 2’ fallacy (Westreich & Greenland, 2013). For the sub‐set of the sample who reported self‐harm, we performed linear regressions with the same socio‐demographic and environmental factors as independent variables and the age of initiation of self‐harm as the dependent variable.

To investigate factors associated with motivations and help‐seeking behaviour, five sets of linear regression analyses were conducted within the sub‐set of the sample who reported self‐harm. The age of initiation was an independent (or predictor) variable in all models, alongside the socio‐demographic and environmental factors (SES, female gender, gender minority status, sexual minority status, and lifetime victimisation) as covariates, and the rating on each motivation to self‐harm as dependent variables. Furthermore, a logistic regression was conducted with the age of initiation as the independent variable, alongside the socio‐demographic and environmental factors (same as above), as covariates, and help‐seeking as the dependent variable. For all the regression models, clustered standard errors were applied to set the variance in the errors to constant within clusters (twin pairs), and thus control for the non‐independence of twins when estimating standard errors. Listwise deletion was applied in all the phenotypic models to handle missing data.

#### Genetic analyses

To address the third aim, we applied the classical twin design, which compares within‐twin pair correlations in MZ and DZ twins. A comprehensive description of twin data analysis can be found elsewhere (Rijsdijk & Sham, 2002). Univariate analyses were conducted to quantify the heritability of self‐harm at ≤16, 21, and 26 in the TEDS sample, by decomposing the observed variance in self‐harm into additive genetic effects (A), shared environmental effects (C), and non‐shared environmental effects (E). ACE and AE models were compared with the saturated model using raw data maximum likelihood, with log likelihood (−2LL) used as a relative measure of fit. Liability threshold univariate modelling (Sham et al., 1994) was applied due to the self‐harm data being ordinal, using the twin concordance rates and full‐information maximum likelihood. To fit all the genetic models, structural equation modelling (Boker et al., 2011) was applied, using OpenMx (Neale et al., 2016) – a package in R (R Core Team, 2012) designed for analysing twin data.

We also fitted a trivariate Cholesky decomposition model to test stability and innovation in genetic and environmental influences from ≤16 to 26. This model specified self‐harm as a liability threshold variable. The Cholesky decomposition partitions A, C, and E components into three sets of factors acting at time 1 (T1), time 2 (T2), and time 3 (T3): AT1, CT1, and ET1 act on all three variables, AT2, CT2, and ET2 act on the second (age 21) and third (age 26) variables and AT3, CT3, and ET3 act only on the third (age 26) variable. Inferences about the direction of effects could be drawn due to the temporal ordering of the variables (Rijsdijk & Sham, 2002). In all of the genetic models, missing data were handled using full‐information maximum likelihood estimates.

Our analytical plan was pre‐registered on Open Science Framework (https://osf.io/yp95a). An edit was made to the transformation of the sexual minority status variable to use reported sexual orientation at 26 instead of sexual attraction reported at 21 due to better construct validity.

## Results

### Descriptive statistics

The median and modal ages of initiation of self‐harm were 15 and 14 years, respectively. The rate of self‐harm initiation between 17 and 21 years was 11.6% (*N* = 904), and between 22 and 26 years, it was 1.5% (*N* = 91). The prevalence (past 12 months) was 10.4% (*N* = 881) at age 21, and 8.9% at age 26 (*N* = 694). The lifetime prevalence of self‐harm was 17.7% (*N* = 1,660) at age 16 and 25.7% (*N* = 2,412) at 21. Participants who reported self‐harm at 21 were subsequently asked how often they self‐harmed without and with suicidal intent – NSSH was reported by 86.2% (*N* = 2060) and SSH by 42.3% (*N* = 1,011); 39.3% (*N* = 932) reported both SSH and NSSH. The rates of participants reporting different frequencies of lifetime self‐harm at age 21 can be found in Table S3. The most commonly reported motivations were ‘to get relief from a terrible state of mind’, reported by 83.4% of the participants who self‐harmed, and ‘to punish oneself’ (reported by 70.7%). A minority (32.3%) sought professional help after self‐harming (See Table S4 for more details).

### Group differences in self‐harm and its age of initiation

There were no significant differences between the racialised minority and majority in self‐harm and the age of initiation. There were significant group differences between gender and sexual minorities in self‐harm at ≤21 years and in the age of initiation of self‐harm (see Table 1 for statistics). With regards to the lifetime self‐harm at age 21, sensitivity analyses showed that using a continuous self‐harm score instead of dichotomous did not affect the results (See Table S5).

**Table 1:** Results of Chi‐square test for group differences

Dependent variable	Group comparison	*df*	*N*	*χ* ^2^	*p*
Lifetime self‐harm at age 21	Racialised minority group	1	9,327	2.21	.14
Lifetime self‐harm at age 21	Genders	2	6,553	153.35***	<.001
	Sexual orientations	4	6,324	350.94***	<.001
Age of initiation of self‐harm	Racialised minority group	1	2,312	7.52	.38
Age of initiation of self‐harm	Genders	14	1,709	68.57***	<.001
	Sexual orientations	28	1,621	50.22**	<.01

*Note*: Racialised minority group is compared with the racialised majority; Analyses for genders compare female, male, and non‐binary/gender‐queer; Analyses for sexual orientations compare heterosexual, homosexual, bisexual, pansexual, and asexual.

*p = .05, **p = .01, ***p = .001.

### Regression models

#### Associations between socio‐demographic factors, lifetime victimisation, self‐harm, and the age of initiation

The mean lifetime peer victimisation score was 5.48 (*SD* = 4.37). Only 3.6% of the participants reported no lifetime peer victimisation. Univariate logistic and linear regression models were used to quantify the association between self‐harm at ≤21 (models 1 and 2), the age of initiation (model 3), and the following predictor variables: SES, female gender, gender minority status, sexual minority status, and lifetime peer victimisation. Due to the lack of differences in self‐harm and age of initiation between racialised minority and majority individuals, racialised minority status was not included as an independent variable. Clustered standard errors were applied to account for non‐independence of the data. Female gender, gender minority status, sexual minority status, and lifetime victimisation were consistently associated with greater odds (dichotomous outcome; OR = 1.82, 5.43, 3.64, 1.98, respectively) and rates (continuous outcome; *B* = 0.29, 1.37, 0.89, 0.34, respectively) of lifetime self‐harm at ≤21 years and with earlier age of initiation of self‐harm (*B* = −0.83, −0.66, −0.46, −0.43, respectively) in unadjusted models (see Table 2). Lower SES was significantly associated with greater odds and rates of self‐harm (OR = 0.88, and *B* = −0.04), but not with earlier initiation (*B* = −0.02). Adjusted models, including all covariates were also estimated and followed a similar pattern of results (see Table S6). A sensitivity analysis was conducted for the first and second models, where self‐harm at age ≤21 and self‐harm at age 26 were combined, and the pattern of results was largely the same (see Table S7).

**Table 2:** Statistics of logistic and linear regression models predicting self‐harm at ≤21 and age of initiation of self‐harm

	*N*	Unadjusted *B* (95% CI)	Beta *SE*	Odds ratio (95% CI)	*p*
Predicting self‐harm at age ≤21 (Dichotomous measure)					
SES	8,637	−0.13*** (−0.18, −0.08)	0.05	0.88 (0.83, 0.93)	<.01
Female gender	6,498	0.60*** (0.47, 0.73)	0.11	1.82 (1.60, 2.08)	<.001
Gender minority status	6,558	1.69*** (1.24, 2.14)	1.03	5.43 (3.47, 8.49)	.32
Sexual minority status	6,397	1.29*** (1.15, 1.43)	0.11	3.64 (3.16, 4.19)	<.001
Lifetime bullying victimisation	4,408	0.68*** (0.58, 0.78)	0.01	1.98 (1.79, 2.18)	<.001
Predicting self‐harm at age ≤21 (Continuous measure)					
SES	8,637	−0.04*** (−0.07, −0.02)	0.01		.09
Female gender	6,498	0.29*** (0.23, 0.34)	0.03		<.001
Gender minority status	6,558	1.37*** (1.01, 1.73)	0.18		<.05
Sexual minority status	6,397	0.89*** (0.79, 1.00)	0.05		<.001
Lifetime bullying victimisation	4,408	0.34*** (0.29, 0.40)	0.00		<.001
Predicting age of initiation of self‐harm					
SES	2,123	−0.02 (−0.11, 0.08)	0.05		.35
Female gender	1,670	−0.83*** (−1.07, −0.58)	0.12		<.001
Gender minority status	1,713	−0.66* (−1.21, −0.12)	0.28		<.05
Sexual minority status	1,660	−0.46*** (−0.69, −0.23)	0.12		<.05
Lifetime bullying victimisation	1,018	−0.43*** (−0.59, −0.27)	0.01		<.001

In the logistic regression (dichotomous treatment of the self‐harm measure), the *B* coefficient is the log of the odds ratio – for continuous predictors odd ratios compare individuals who differ by one unit of a predictor, whereas for categorical predictors it compares individuals at a particular level of the predictor to a reference level; Unadjusted estimates reflect simple regression models where each variable is a sole predictor variable.

*p = .05, **p = .01, ***p = .001.

Post hoc analyses (not pre‐registered) were conducted to investigate if the associations persist after controlling for familial factors. Co‐twin control (for binary exposures) and MZ differences (for continuous exposures) designs were applied (described in McAdams, Rijsdijk, Zavos, & Pingault, 2021). These were tested for the variables that can conceptually be unshared between twins (sexual minority status and bullying victimisation). The association persisted between sexual minority status and lifetime self‐harm reported at 21 (*B* 95% CI = 0.11–0.98), but not between victimisation and self‐harm (*B* 95% CI = −0.22 to 0.45). See Table S8 for details. Gender minority status may be unshared between MZ twins, but it was not tested due to a small number (*N* = 29) of MZ twins reporting to be transgender or non‐binary.

#### Factors associated with motivations for self‐harm and help‐seeking

Earlier age of initiation was associated with being motivated to self‐harm to ‘show how desperate [one] was feeling’, ‘die’, ‘punish oneself’, and ‘frighten someone’. SES was negatively associated with motivation to self‐harm ‘to die’ (see Table 3). Female gender was positively associated with being motivated to self‐harm to ‘punish oneself’ and ‘get relief from a terrible state of mind’. Gender minority status was positively associated with being motivated to self‐harm to ‘die’ and ‘get relief from a terrible state of mind’. Sexual minority status was positively associated with being motivated to self‐harm in order to ‘die’, ‘punish oneself’, and ‘get relief from a terrible state of mind’. Lifetime victimisation was positively associated with being motivated to self‐harm to ‘show how desperate [one] was feeling’, ‘die’, ‘punish oneself’, ‘frighten someone’, and ‘get relief from a terrible state of mind’.

**Table 3:** Statistics of linear regression models predicting different motivation for self‐harm

	Independent variables						
	Age of initiation	Racialised minority status	SES	Female gender	Gender minority status	Sexual minority status	Lifetime victimisation
	Unadjusted *B* (*SE*)						
‘to show how desperate you were feeling’	−0.04*** (0.01)	0.19 (0.12)	0.03 (0.03)	0.14 (0.08)	0.15 (0.17)	−0.10 (0.07)	0.13 (0.05)**
‘to die’	−0.07*** (0.01)	0.05 (0.13)	−0.09 (0.03)**	0.00 (0.08)	0.68 (0.20)**	0.48 (0.08)***	0.32 (0.06)***
‘to punish oneself’	−0.08 (0.01)***	0.14 (0.13)	0.05 (0.03)	0.30 (0.08)***	0.54 (0.16)***	0.43 (0.08)***	0.30 (0.05)***
‘to frighten someone’	−0.02 (0.01)*	0.11 (0.08)	0.00 (0.02)	0.05 (0.05)	−0.13 (0.11)	−0.07 (0.05)	0.14 (0.03)***
‘to get relief from a terrible state of mind’	0.02 (0.01)	−0.09 (0.12)	−0.03 (0.03)	0.31 (0.08)***	0.67 (0.11)***	0.40 (0.07)**	0.18 (0.05)***

Models are reported for a sub‐sample of twins who reported lifetime self‐harm at 21, and each motivation (e.g. ‘to die’) was a dependent variable in a separate model with the same independent variables for each of these models.

*p < .05, **p < .01, ***p < .001.

Age of initiation, ethnic minority status, female gender, and SES were not associated with help‐seeking, whilst gender minority status, sexual minority status, and lifetime victimisation were significantly associated with greater likelihood of seeking professional help. See Table 4 for detailed statistics.

**Table 4:** Statistics of logistic regression models predicting help‐seeking behaviour

	Independent variables						
	Age of initiation	Ethnic minority status	SES	Female gender	Gender minority status	Sexual minority status	Lifetime victimisation
	Undjusted *B* (*SE*)						
Sought professional help	0.02 (0.02)	0.07 (0.19)	−0.06 (0.05)	0.22 (0.12)	0.56 (0.26)*	0.50 (0.11)***	0.28 (0.00)***

Models are reported for a sub‐sample of twins who reported lifetime self‐harm at 21.

*p < .05, **p < .01, ***p < .001.

### Aetiology of self‐harm

#### Univariate results

Components of variance in each phenotype were estimated and the best‐fitting models were inferred, for each variable. Genetic effects (A) were moderate and comparable for self‐harm at age ≤16 (0.54; 95% CI 0.47–0.61), age 21 (0.51; 95% CI 0.34–0.60), and age 26 (0.44; 95% CI 0.06–0.57). Non‐shared environmental effects (E) explained the remaining proportion of the variance and were similar across the different timepoints. For all variables, shared environmental effects (C) were non‐significant. See Table S9 for detailed standardised variance components as well as MZ and DZ twin correlations.

#### Multivariate results

Self‐harm was found to be moderately correlated across the three time points, indicating that multivariate models were warranted (See Table S10 for more detail).

**Figure 1:**
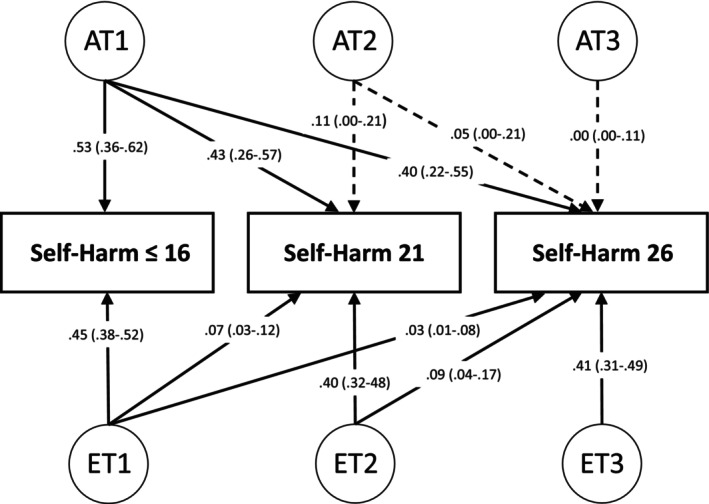
Trivariate Cholesky decomposition twin model for self‐harm at ages ≤16, 21, and 26, displaying shared and specific A and E variance components and 95% CIs

A trivariate Cholesky model provided a good fit for the data when compared with the saturated model (−2LL = 16,410.35, Δ−2LL = 39.92, Δ*df* = 30, *p* = .11). Parameter estimates for A and E shared and specific paths are presented in Figure 1, as none of the C estimates were significant (See Table S11). Total A, C, and E estimates on each time point can be obtained by summing the contributions of common and specific elements, for example, at age 26 AT1 is .40, AT2 is .05, and AT3 is .00, which sums up to .45. These were found to be generally consistent with the A, C, and E components derived from the univariate models (Table S9). Results showed a stable genetic factor (AT1) which influenced self‐harm at all three time points, and innovation in non‐shared environmental factors (ET1, ET2, ET3). The AT1 factors significantly accounted for 53% of the variance in self‐harm at time 1, whilst also accounting for 43% of the variance at time 2 and 40% of variance at time 3, whereas AT2 and AT3 factors had non‐significant influence on time 2 and time 3. On the other hand, the largest environmental proportion of variance was consistently explained by concurrent non‐shared environmental effects, that is, 45% of time 1 variance was explained by ET1 (time 1 non‐shared environmental effects), 40% of the time 2 variance was explained by ET2, and 41% of the time 3 variance was explained by ET3.

## Discussion

This study found that in line with previous research (Carr et al., 2016; Plener et al., 2015), self‐harm is more common in adolescence, and declines in early adulthood. Results suggested that it was unusual for self‐harm to be initiated between the ages of 22 and 26 – self‐harming at age 26 with no history of self‐harm in adolescence and early 20s had a very low rate. Most young people initiated before the age of 17 (17.7%), but the rate of initiation between ages 17 and 21 was still high (11.6%). This suggests that adolescence is a crucial time to target interventions around self‐harm. Within the broad definition of self‐harm, non‐suicidal intent was more common than suicidal intent, although suicidal self‐harm was still reported by over 40% of those who self‐harmed before age 21. Women, sexual and/or gender minorities, those growing up in lower SES households, and those who had experienced bullying victimisation were more likely to have self‐harmed, in line with previous research (Gillies et al., 2018; Page et al., 2014; Plener et al., 2018; Oginni et al., 2019, Rogers & Taliaferro, 2020). Sexual minority youth were still more likely to self‐harm when accounting for confounding familial vulnerabilities in a co‐twin control analysis. This extends previous research, which observed the same in an adolescent sample (O'Reilly, Pettersson, Donahue, et al., 2021), by replicating it in a young adult sample. We were able to further previous research by showing that all these factors, except SES, were also linked to an earlier age of initiation of self‐harm. Cholesky decomposition indicated that environmental influences accounted for roughly half the variance in self‐harm at each time point, and new sources of environmental effects emerged across adolescence and early adulthood, whilst genetic influences remained stable. The heritability across adolescence and early adulthood was moderate, which is in line with previous estimates for young adulthood self‐harm (Lim et al., 2022).

Our findings suggest that female participants as well as sexual and gender minority groups have higher rates of self‐harm and initiate self‐harm earlier than males and members of sexual and gender majority groups. It might be that adolescent girls are more likely to initiate self‐harming due to gender inequalities, some of which also relate to patriarchal gendered socialisation (being taught to behave in line with the social expectations of one's gender roles). For instance, girls and women are more likely to experience violence and abuse and expectations to work without pay (Shute, 2016), as well as more likely to be objectified and sexualised in the media, including social media (e.g. Hartas, 2021; Kelly, Zilanawala, Booker, & Sacker, 2018). However, more research is needed on the mechanisms of gender differences in self‐harm. As we also found victimisation to be associated with self‐harm, victimisation could be a mechanism through which minority groups develop higher self‐harm rates. To explore this further, we conducted a public engagement consultation for this project with an Adolescent Advisory Group at King's College London comprising seven adolescents aged 14–19 years. The group highlighted that those not fitting social norms are often bullied for being different. However, in the present study, the association between victimisation and self‐harm was relatively weaker than between some of the other predictors and self‐harm (i.e. sexual and gender minority statuses). The advisory group proposed that bullying is very common, which could be an explanation for this in that many adolescents experience victimisation but only some go on to self‐harm. Furthermore, victimisation on the basis of gender and sexual minority statuses was not directly measured. However, it seems that other factors play a greater role in the initiation of self‐harm. For instance, minority groups might develop self‐harm through marginalisation and micro‐aggressions that are not specified as bullying victimisation, which still triggers difficult emotions (Frost & Meyer, 2023). Considering the most common motivations for self‐harm found in this study (i.e. relief, self‐punishment) and their associations with sexual and gender minority statuses, minority stress is a plausible explanation for these findings.

### Clinical and research implications

The impact of this study is the insight into risk factors (e.g. socio‐demographic), characteristics (i.e. motivation for self‐harm and help‐seeking behaviours) of self‐harm, and its early initiation, indicating potential avenues for prevention strategies. We extend the findings on the average age of initiation, to show which characteristics put adolescents at a higher likelihood of initiating self‐harm early. Prevention strategies could target girls, LGBTQ+ adolescents, and those experiencing bullying starting in early adolescence (ages 11–14). The findings on motivations and help‐seeking also indicate a potential avenue for intervention – most young people report self‐harming to ‘get relief from a terrible state of mind’ and most do not seek help. It suggests that self‐harm might be a way of attempting to cope with psychological distress, which has been evidenced directly in past research (e.g. Mikolajczak, Petrides, & Hurry, 2009). Teaching other coping strategies in adolescence and improving access to psychological support might decrease distress and the likelihood of self‐harm. Destigmatising initiatives, such as educating healthcare professionals and offering information pamphlets about self‐harm, might increase help‐seeking following self‐harm, which might lead to increased referrals to psychological support. Finally, the evidence that genetic risk for self‐harm remains stable whilst new environmental factors emerge highlights the importance of environmental interventions to prevent self‐harm and the outcomes associated with it, such as death by suicide. Furthermore, the persistent genetic vulnerability to self‐harm may be explained by stable liability to psychiatric condition, as previous research showed that genetic effects on the major depressive disorder, attention‐deficit/hyperactivity disorder, and schizophrenia predicted self‐harm (Lim et al., 2020); therefore, interventions addressing symptoms of these conditions may play a role in preventing self‐harm.

### Strengths and limitations

The present study has several strengths and limitations that are important to consider. One of the strengths is the investigation of sexual and gender minority status as risk factors in a representative sample of the population studied. This means that the results may be generalisable to the larger population. However, there are also several limitations. One limitation is the retrospective manner in which the self‐harm measure at age ≤16 was generated. It would be more reliable to test the aetiology of self‐harm at age ≤16 using a prospective report, which should be done in future studies. The study's findings on factors associated with self‐harm and its early initiation are not necessarily causal, and there is a possibility of reverse causality or confounding. Furthermore, phenotypic and genetic findings from the study may not be generalisable to other age groups, as self‐harm differs in rates and presentation across different ages (e.g. McManus et al., 2019). The findings may not be generalisable due to the characteristics and selection bias of the TEDS sample. For example, whilst TEDS was representative of the population at the time of first contact, the racialised minority population is now larger, even in the same generation as TEDS twins (i.e. young adults of racialised background of the same age who moved to England and Wales but were not born there). This is a significant consideration as 13.5% of 16–24‐year‐olds in the UK in 2011 were non‐UK‐born (The Migration Observatory, 2015). Future research could use other, non‐birth cohorts to study young people's self‐harm. Furthermore, the way that minoritisation (on the base of race, gender, or sexuality) was used in the analysis, due to power constraints, overlooks the heterogeneity of minoritised groups. The classical twin design used in the study also has limitations (Røysamb & Tambs, 2016). Future studies should replicate the early initiation findings in the general, non‐twin population.

## Conclusions

In conclusion, in this study conducted in a UK population‐based sample of twins who are now in their late 20s, we found evidence that self‐harm is more common in adolescence than early adulthood, making it a key period to intervene. Furthermore, we found that previously evidenced predictors of self‐harm also predict its earlier initiation. Help‐seeking following self‐harm is low, and both help‐seeking and motivations for self‐harm are unrelated to the age of initiation. Different individuals can be at risk at different stages as environmental factors influencing self‐harm change across time, whilst genetic factors remain stable.

**Key points**

- Self‐harm is a common behaviour in adolescence and early adulthood with most young people initiating self‐harm in early adolescence.
- Our findings showed that lower SES, female gender, being a gender and a sexual minority and exposure to bullying victimisation are associated with self‐harm, in line with previous research.
- Further to previous research, we found that broadly the same social factors were also associated with earlier initiation of self‐harm.
- The present study extended previous research which focused on the heritability of self‐harm by showing that genetic influences contribute to the continuity of self‐harm behaviours. Non‐shared environmental influences tended to contribute to change in the behaviours. This highlights the importance of time‐specific environmental interventions.

## Supporting information


**Table S1.** Descriptive statistics of the sample.
**Table S2.** Statistics of logistic regression models predicting attrition at 21 and 26.
**Appendix S1.** Lifetime victimisation measures in TEDS.
**Table S3.** Frequencies of different amounts of lifetime self‐harm at age 21.
**Table S4.** Frequencies of different motivations for self‐harm and for help‐seeking behaviour.
**Table S5.** Results of Chi‐square test for group differences using continuous self‐harm scores (sensitivity analysis).
**Table S6.** Statistics of adjusted logistic and linear regression models predicting self‐harm at ≤21 and age of initiation of self‐harm.
**Table S7.** Statistics of linear and logistic regression models predicting self‐harm at ≤21 and at 26 combined (sensitivity analysis).
**Table S8.** Results of GEE models estimating associations between exposures and binary self‐harm ≤21 when accounting for familial vulnerabilities (MZ differences).
**Table S9.** MZ and DZ cross twin within trait correlations and A, C, and E estimates.
**Table S10.** Phenotypic tetrachoric correlations between self‐harm at different timepoints, *r*.
**Table S11.** Parameter estimates (95% CI) for multivariate longitudinal genetic models of self‐harm between time 1, 2 and 3.

## Data Availability

The data that support the findings of this study are available from TEDS (Twins Early Development Study). Restrictions apply to the availability of these data, but it can be accessed by researchers upon request and the access policy is outlined at https://www.teds.ac.uk/researchers/teds‐data‐access‐policy.
