# Supplementary material for: Initiation and stability of self‐harm in adolescence and early adulthood: investigating social and aetiological factors in twins
Source: J Child Psychol Psychiatry. 2024 Dec 13;66(6):857–67. doi: 10.1111/jcpp.14096 (PMC12062846; doi:10.1111/jcpp.14096)
Supplement: Supplementary file 1 — Table S1. Descriptive statistics of the sample. Table S2. Statistics of logistic regression models predicting attrition at 21 and 26. Appendix S1. Lifetime victimisation measures in TEDS. Table S3. Frequencies of different amounts of lifetime self‐harm at age 21. Table S4. Frequencies of different motivations for self‐harm and for help‐seeking behaviour. Table S5. Results of Chi‐square test for group differences using continuous self‐harm scores (sensitivity analysis). Table S6. Statistics of adjusted logistic and linear regression models predicting self‐harm at ≤21 and age of initiation of self‐harm. Table S7. Statistics of linear and logistic regression models predicting self‐harm at ≤21 and at 26 combined (sensitivity analysis). Table S8. Results of GEE models estimating associations between exposures and binary self‐harm ≤21 when accounting for familial vulnerabilities (MZ differences). Table S9. MZ and DZ cross twin within trait correlations and A, C, and E estimates. Table S10. Phenotypic tetrachoric correlations between self‐harm at different timepoints, r. Table S11. Parameter estimates (95% CI) for multivariate longitudinal genetic models of self‐harm between time 1, 2 and 3. [file JCPP-66-857-s001.docx]

**Supporting Information:**

**Table S1**. Descriptive Statistics of the Sample

| **Characteristics** |  |
| --- | --- |
|  | **Reported in Mean (SD)** |
| **SES Indices** |  |
| **Maternal Education (1-8 scale)** | 3.88 (2.01) |
| **Maternal Occupation (1-9 scale)** | 3.92 (2.20) |
| **Parental Education (1-8 scale)** | 4.12 (2.25) |
| **Parental Occupation (1-9 scale)** | 3.82 (2.56) |
|  | **Reported in % and (*n*)** |
| **Birth sex at first contact (female)** | 53.8% (9226) |
| **Gender identity at age 26 (woman)** | 64.5% (5271) |
| **Non-binary/genderqueer** | 0.7% (61) |
| **Transgender** | 0.6% (51) |
| **Gender Minority Status**(i.e., non-binary or trans) |  |
| **Gender Minority** | 1.4% (112) |
| **Sexual Orientation** |  |
| **Straight** | 78.9% (7522) |
| **Gay/lesbian** | 11.3% (1080) |
| **Bisexual** | 2.0% (189) |
| **Pansexual** | 2.0% (186) |
| **Asexual** | 3.5% (337) |
| **Fluid** | 1.4% (138) |
| **Self-defined** | 0.8% (78) |
| **Sexual Minority Status** |  |
| **Sexual Minority** | 14.8% (1167) |
| **Ethnicity** |  |
| **English, Welsh, Scottish, Northern Irish or British** | 92.7% (7593) |
| **Irish** | 0.4% (35) |
| **Any other white background** | 0.9% (75) |
| **White and black Caribbean** | 1.0% (85) |
| **White and Asian** | 0.8% (66) |
| **Any other mixed ethnic background** | 0.7% (58) |
| **Indian** | 1.2% (102) |
| **Pakistani** | 0.6% (47) |
| **Chinese** | 0.2% (15) |
| **Any other Asian background** | 0.3% (25) |
| **African** | 0.3% (23) |
| **Caribbean** | 0.4% (35) |
| **Any other ethnic group** | 0.3% (25) |
| **Ethnic Minority Status** |  |
| **Ethnic Minority** | 7.3% (1232) |

**Table S2**. Statistics of Logistic Regression Models Predicting Attrition at 21 and 26

|  | *Beta* | *S.E.* | 95% CI | | *p* |
| --- | --- | --- | --- | --- | --- |
|  |  |  | ***LL*** | ***UL*** |  |
| Predicting Response to TEDS 21 Phase 1 | | | | | |
| Self-harm age ≤16 | 0.00 | 0.03 | -.06 | .06 | 1.00 |
| Predicting Response to TEDS 21 Phase 2 | | | | | |
| Self-harm age ≤16 | 0.17* | 0.08 | .02 | .33 | 0.03 |
| Predicting Response to TEDS 26 | | | | | |
| Self-harm age ≤16 | 0.24*** | 0.06 | .11 | .36 | <.001 |
| Self-harm age ≤21 | 0.12*** | 0.02 | .07 | .16 | <.001 |

*Note:* * *p* = .05, ** *p* = .01, *** *p* = .001; In logistic regressions, the *B* coefficient is the log of the odds ratio – for continuous predictors odd ratios compare individual who differ by one unit of a predictor, whereas for categorical predictors it compares individuals at a particular level of the predictor to a reference level

**Appendix S1.** Lifetime Victimisation Measures in TEDS

TEDS 12 and 14 used the following items from Multidimensional Peer Victimisation Scale (MPVS; Mynard & Joseph, 2000), which were scored as “not at all”, “once”, “more than once”:

[How often during the last year has someone (excluding family and partner) done these things to you?]

1. Punched me

2. Tried to get me into trouble with my friends

3. Called me names

4. Took something of mine without permission

5. Kicked me

6. Tried to make my friends turn against me

7. Made fun of me because of my appearance

8. Tried to break something of mine

9. Hurt me physically in some way

10. Refused to talk to me

11. Made fun of me for some reason

12. Stole something from me

13. Beat me up

14. Made other people not talk to me

15. Swore at me

16. Deliberately damaged some property of mine

TEDS 16 used the following items from a shortened version of the MPVS, with 6-items only, which were scored as “not at all”, “once”, “more than once”:

[How often during the last year has someone (excluding family and partner) done these things to you?]

1. Tried to get me into trouble with my friends

2. Took something of mine without permission

3. Hurt me physically in some way

4. Refused to talk to me

5. Made fun of me for some reason

6. Swore at me

TEDS 21 used the following items adapted from the Multidimensional Peer-Victimisation Scale-Revised (MPVS-R; Betts, Houston, & Steer, 2015), which were scored as “not at all”, “once”, “more than once”:

[How often during the last year has someone (excluding family and partner) done these things to you?]

1. Punched me

2. Tried to get me into trouble with my friends 

3. Called me names

4. Sent me nasty texts

5. Kicked me

6. Tried to turn my friends against me

7. Made fun of me because of my appearance

8. Said something mean about me on social media

1. (e.g., Facebook, Instagram)

9. Hurt me physically in some way

10. Refused to talk to me

11. Made fun of me for some reason

12. Written spiteful things about me in a chat room

13. Beaten me up

14. Made other people not talk to me

15. Sworn at me

16. Written nasty things to me using instant messenger (e.g., Facebook Messenger, Whatsapp, Snapchat)

**Table S3**. Frequencies of Different Amounts of Lifetime Self-Harm at age 21

| Amount of Self-Harm Events | % (n) |
| --- | --- |
| 0 | 74.3% (6989) |
| 1-2 | 12.4% (1168) |
| 3-5 | 4.2% (394) |
| 6-10 | 2.4% (227) |
| >10 | 6.6% (623) |
| Amount of Non-Suicidal Self-Harm Events | |
| 0 | 77.2% (6989) |
| 1-2 | 10.8% (980) |
| 3-5 | 3.4% (307) |
| 6-10 | 2.3% (205) |
| >10 | 6.3% (568) |
| Amount of Suicidal Self-Harm Events | |
| 0 | 87.4% (6989) |
| 1-2 | 8.4% (670) |
| 3-5 | 2.0% (158) |
| 6-10 | 0.8% (62) |
| >10 | 1.5% (121) |

**Table S4**. Frequencies of Different Motivations for Self-Harm and for Help-Seeking Behaviour

| **Motivation for Self-Harm** | **% (n/N)** |
| --- | --- |
| To show how desperate you were feeling | 46.8% (1112/2376) |
| To die | 40.4% (963/2383) |
| To punish oneself | 70.7% (1693/2393) |
| To frighten someone | 12.8% (307/2398) |
| To get relief from a terrible state of mind | 83.4% (1997/2394) |
| **Sought professional help** |  |
| From any healthcare professional | 32.3% (765/2369) |
| GP | 25.8% (609/2365) |
| Hospital | 12.1% (287/2367) |
| Other healthcare provider | 14.3% (338/2362) |

*Note*: n denotes the number of participants who reported said motivation or help-seeking, whereas N denotes the total number of participants who answered the question

**Table S5**. Results of Chi-square Test for Group Differences Using Continuous Self-Harm Scores (Sensitivity Analysis)

| Dependent Variable | Group Comparison | *df* | *N* | *X^2^* | *p* |
| --- | --- | --- | --- | --- | --- |
| Lifetime self-harm at 21 | Racialised group | 4 | 9,327 | 3..39 | .50 |
|  | Genders | 8 | 6,553 | 209.13 | <.001 |
|  | Sexual orientations | 16 | 6,325 | 515.08 | <.001 |

*Note:* Racialised Minority Group is compared to the Racialised Majority; Analyses for genders compare female, male, and non-binary/gender-queer; Analyses for sexual orientations compare heterosexual, homosexual, bisexual, pansexual, and asexual.

**Table S6**. Statistics of Adjusted Logistic and Linear Regression Models Predicting Self-Harm at ≤ 21 and Age of Initiation of Self-Harm

|  | Adjusted *B* (95% CI) | *Beta* S.E. | Odds Ratio (95% CI) | *p* |
| --- | --- | --- | --- | --- |
| Predicting Self-Harm at age ≤21 (Dichotomous Measure), *N* = 3,101 | | | | |
| SES | -0.16** (0.00, -0.26) | 0.05 | 0.08 (0.06, 0.11) | <.01 |
| Female gender | 0.87*** (0.65, 1.09) | 0.11 | 2.38 (1.91, 2.96) | <.001 |
| Gender Minority Status | 1.21 (-0.80, 3.22) | 1.03 | 3.35 (0.45, 24.98) | 0.32 |
| Sexual Minority Status | 1.20*** (0.98, 1.42) | 0.11 | 3.31 (2.65, 4.13) | <.001 |
| Lifetime Bullying Victimisation | 0.10*** (0.08, 0.12) | 0.10 | 1.11 (1.09, 1.13) | <.001 |
| Predicting Self-Harm at age ≤21 (Continuous Measure), *N* = 3,101 | | | | |
| SES | -0.04 (-0.08, 0.01) | 0.02 |  | .09 |
| Female gender | 0.34*** (0.27, 0.42) | 0.04 |  | <.001 |
| Gender Minority Status | 1.43* (0.02, 2.84) | 0.72 |  | <.05 |
| Sexual Minority Status | 0.77*** (0.62, 0.91) | 0.08 |  | <.001 |
| Lifetime Bullying Victimisation | 0.05*** (0.04, 0.06) | 0.01 |  | <.001 |
| Predicting Age of Initiation of Self-Harm, *N* = 720 | | | | |
| SES | -0.08 (-0.24, 0.09) | 0.08 |  | .35 |
| Female gender | -0.86*** (-1.23, -0.50) | 0.19 |  | <.001 |
| Gender Minority Status | -1.89* (-3.40, -0.40) | 0.77 |  | <.05 |
| Sexual Minority Status | -0.46* (-0.81, -0.11) | 0.18 |  | <.05 |
| Lifetime Bullying Victimisation | -0.06*** (-0.10, -0.03) | 0.02 |  | <.001 |

*Note*: * *p* = .05, ** *p* = .01, *** *p* = .001; In the logistic regression (dichotomous treatment of the self-harm measure), the *B* coefficient is the log of the odds ratio – for continuous predictors odd ratios compare individual who differ by one unit of a predictor, whereas for categorical predictors it compares individuals at a particular level of the predictor to a reference level; Adjusted estimates adjust for the effect of the covariates

**Table S7**. Statistics of Simple Linear and Logistic Regression Models Predicting Self-Harm at ≤ 21 & at 26 combined (Sensitivity Analysis)

|  | *Undjusted B (95% CI)* | *Beta S.E.* | Odds Ratio (95 % CI) | *p* |
| --- | --- | --- | --- | --- |
| Predicting Self-Harm at ages ≤21 and 26 (binary measure) | | | | |
| SES | -0.04*** (-0.05, -0.03) | 0.01 | 0.96 (0.95, 0.97) | <.001 |
| Female gender | 0.12*** (0.10, 0.14) | 0.01 | 1.13 (1.10, 1.15) | <.001 |
| Gender Minority Status | 0.45*** (0.36, 0.55) | 0.05 | 1.58 (1.44, 1.73) | <.001 |
| Sexual Minority Status | 0,31*** (0.28, 0.35) | 0.02 | 1.37 (1.32, 1.41) | <.001 |
| Lifetime Peer Victimisation | 0.16*** (0.14, 0.18) | 0.01 | 1.17 (1.15, 1.20) | <.001 |

| Predicting Self-Harm at ages ≤21 and 26 (continuous measure) | | | | |
| --- | --- | --- | --- | --- |
| SES | -0.07*** (-0.11, -0.03) | 0.02 | 0.93 (0.89, 0.97) | <.001 |
| Female gender | 0.36*** (0.29, 0.44) | 0.04 | 1.44 (1.33, 1.55) | <.001 |
| Gender Minority Status | 2.21*** (1.62, 2.79) | 0.30 | 9.08 (5.05, 16.33) | <.001 |
| Sexual Minority Status | 1.15*** (1.01, 1.30) | 0.07 | 3.17 (2.74, 3.67) | <.001 |
| Lifetime Peer Victimisation | 0.52*** (0.42, 0.61) | 0.05 | 1.68 (1.53, 1.84) | <.001 |

*Note*: * *p* = .05, ** *p* = .01, *** *p* = .001; For continuous predictors odd ratios compare individual who differ by one unit of a predictor, whereas for categorical predictors it compares individuals at a particular level of the predictor to a reference level. In the logistic regression (dichotomous treatment of the self-harm measure), the *B* coefficient is the log of the odds ratio; Unadjusted estimates reflect simple regression models where each variable is a sole predictor variable

**Table S8.** Results of GEE models estimating associations between exposures and binary self-harm ≤21 when accounting for familial vulnerabilities (MZ differences)

| Exposure | Total *N* MZ Twins (*N* reporting the Exposure*) | *Unadjusted B Coefficient (95% CI)* | *S.E.* |
| --- | --- | --- | --- |
| Sexual Minority Status | 1,458 (209) | 0.55 (0.11, 0.98) | 0.05 |
| Bullying Victimisation | - | 0.11 (-0.22, 0.45) | 0.17 |

*Note*: **N* reporting the outcome is only relevant and reported for the binary measures, hence only reported for sexual minority status

**Table S9.** MZ and DZ Cross Twin Within Trait Correlations and A, C, and E Estimates

| Variable | rMZ (95% CI) | rDZ (95% CI) | A (95% CI) | C (95% CI) | E (95% CI) |
| --- | --- | --- | --- | --- | --- |
| Self-Harm ≤ 16 | .55 (.46-.62) | .26 (.18-.34) | .54 (.47-.61) | .00 (.00-.16) | .46 (.39-.53) |
| Self-Harm 21 | .54 (.43-.64) | .16 (.04-.28) | .51 (.34-.60) | .00 (.00-.11) | .49 (.40-.60) |
| Self-Harm 26 | .45 (.29-.59) | .21 (.06-.35) | .44 (.06-.57) | .00 (.00-.29) | .56 (.43-.70) |

*Note:* rMZ = cross-twin within-trait correlation for monozygotic twins; rDZ = within-pair within-trait correlation for dizygotic twins; A = additive genetic influence; C = shared environmental influence; E = non-shared environmental influence; 95% CI = 95% confidence interval

**Table S10.** Phenotypic Tetrachoric Correlations between Self-Harm at Different Timepoints, *r* (95% CIs)

|  | Self-Harm ≤ 16 | Self-Harm at 21 | Self-Harm at 26 |
| --- | --- | --- | --- |
| Self-Harm ≤ 16 | 1 |  |  |
| Self-Harm at 21 | .66 (.60-.69) | 1 |  |
| Self-Harm at 26 | .59 (.53-.65) | .70 (.66-.76) | 1 |

**Table S11.** Parameter estimates (95% CI) for multivariate longitudinal genetic models of self-harm between time 1, 2 and 3

| Variable | Time 1 Factors | | | Time 2 Factors | | | Time 3 Factors | | |
| --- | --- | --- | --- | --- | --- | --- | --- | --- | --- |
|  | AT1 | CT1 | ET1 | AT2 | CT2 | ET2 | AT3 | CT3 | ET3 |
| Self-Harm ≤ 16 | .53 (.36-.62) | .02 (.00-.11) | .45 (.38-.52) | - | - | - | - | - | - |
| Self-Harm 21 | .43 (.26-.57) | .00 (.00-.13) | .07 (.03-.12) | .11 (.00-.21) | .00 (.00-.09) | .40 (.32-.48) | - | - | - |
| Self-Harm 26 | .40 (.22-.55) | .01 (.00-.17) | .03 (.01-.08) | .05 (.00-.21 | .00 (.00-.00) | .09 (.04-.17) | .00 (.00-.11) | .00 (.00-.09) | .41 (.31-.49) |

*Note:* AT1 = A estimate at time 1, influencing time 2 and 3 as well; CT1 = C estimate at time 1, influencing time 2 and 3 as well; ET1 = C estimate at time 1, influencing time 2 and 3 as well; AT2 = A estimate at time 2, influencing time 3 as well; CT2 = C estimate at time 2, influencing time 3 as well; ET2 = E estimate at time 2, influencing time 3 as well; AT3 = A estimate at time 3, influencing time 3 only; CT3 = C estimate at time 3, influencing time 3 only; ET3 = E estimate at time 3, influencing time 3 only.
